# A journey towards inclusive education; a case study from a ‘township’ in South Africa

**DOI:** 10.4102/ajod.v1i1.15

**Published:** 2012-09-18

**Authors:** Rosemary Luger, Debbie Prudhomme, Ann Bullen, Catherine Pitt, Martha Geiger

**Affiliations:** 1 Chaeli Campaign, Plumstead, South Africa; 2 Centre for Rehabilitation Studies, Stellenbosch University, South Africa

## Abstract

The purpose of this case study was to relate part of the journey to appropriate education for two young children with physical disabilities in a low socio-economic peri-urban informal settlement – or ‘township’ – in South Africa. The part of the on-going journey described here spanned four-and-a-half years and included the two children, their families, their teachers, their community and a small team of rehabilitation professionals working for a non-profit organisation in the area. The rehabilitation professionals’ goals were to provide support for the children, their families, their current special care centre and the school(s) they would attend in the future. The steps from the special care centre, to a mainstream early childhood development (ECD) centre for both of them, and then on to (a) a school for learners with special educational needs (LSEN) for one child and (b) a mainstream primary school for the other, are described. Challenges encountered on the way included parental fears, community attitudes and physical accessibility. Practical outcomes included different placements for the two children with implications and recommendations for prioritised parent involvement, individual approaches, interdisciplinary and community-based collaborations. Recommendations are given for clinical contexts, curricula and policy matters; for research and for scaling up such a programme through community workers.

## Introduction

South Africa is a leader in terms of progressive policies relating to persons with disabilities. In addition to ratifying – and therefore accepting as legally binding – the United Nations convention on the rights of persons with disabilities (United Nations 2008), the principles of inclusive education clearly prescribed in the National Department of Education’s White Paper 6 on inclusive education (Department of Education 2001). In brief, inclusive education is based on the premise that *all* children can learn, and it respects the fact that there are many differences in the ways in which children learn. All children’s educational needs should be met and to do this, attitudes, behaviours, teaching methods and curricula need to be addressed. The focus needs to be on the individual strengths of each child, to maximise participation and minimise barriers to learning (Department of Education 2001; Ogot, McKenzie & Dube 2008; Pillay & Di Terlizzi 2009).

Yet access to inclusive education for children with disabilities in South Africa remains full of diverse challenges (Ogot, McKenzie & Dube 2008; Pillay & Di Terlizzi 2009). For those affected by the additional burden of poverty and lack of resources, this is particularly so.

The purpose of this case study is to recount four years of the journey to appropriate inclusive education for two young children with physical disabilities living in a low socio-economic, peri-urban informal settlement, or ‘township’, in South Africa. In keeping with an ecological systems approach (Garbarino & Ganzel 2000; Pillay & Di Terlizzi 2009), it involved their families, their teachers, their community and a small support team of rehabilitation professionals (which included the authors of this paper). The rehabilitation support team included an occupational therapist, a physiotherapist, a teacher and more recently a community worker, all employed to work part-time in the area by The Chaeli Campaign, a non-profit organisation (NPO). The Chaeli Campaign is actively involved in mobilising children with disabilities in diverse communities across southern Africa. The inclusive education programme with its core focus on sustainable inclusion at early childhood development (ECD) level is one of its six programmes. Our journey with these two children confirmed earlier literature emphasising the need to incorporate a parent- or family-centred approach (Dunst, Bruder, Trivette & Hamby, 2006; Turnbull, Summers, Turnbull *et al*. 2007); to assist the relevant teachers towards a positive attitude to inclusive education and to build strong interdisciplinary collaborations (Silverman, Hong & Trepanier-Street 2010). Here, it resulted in different outcomes for the two children; one child was eventually integrated into a mainstream primary school in his area, while the other child was integrated into a boarding school for learners with special educational needs (an ‘LSEN school’^1^).

## Ethical considerations

This case study falls into the realm of reflective practice (Schön 1995), rather than a formally planned research study. The two children’s stories developed alongside each other, drawing the interventionist team’s attention to the value of sharing the journey with others in this case study paper. The fact that both children described here happen to be boys, is coincidental and not at all representative of the girl-led Chaeli Campaign’s focus,^2^ or the interventionist team’s general client profile.

In order to request consent from the children and their parents to write up this case study, the community worker and the occupational therapist met with each child and one of his parents. A slight adaptation of the Stellenbosch University, Faculty of Medicine and Health Sciences’ Human Research Ethics Consent form for children (translated into Xhosa by the community worker) was used to guide the discussion. Both children and their parents understood the purpose and process of the case study report. Both boys wanted their real names to be used rather than pseudonyms, but details identifying the context were removed in order to keep other role players anonymous and to preserve the confidentiality of some sensitive case information.

### The journey

The two Xhosa boys, part of whose respective journeys is described here, were living with their mothers, the father and an aunt respectively, and one sibling each, in informally constructed dwellings (or ‘shacks’) in a low-socioeconomic, informal settlement (‘township’) in the larger Cape Town area, in South Africa.

Ayabonga was born two months prematurely in 2004. He was slow to develop (e.g. he sat at 14 months and crawled at the age of two years) and was diagnosed with spinal muscular atrophy (SMA) Type 2. This is a rare disease where there is an abnormality of the anterior horn cells in the spinal cord due to a chromosomal defect. Ayabonga is a friendly child who eats independently, is a skilled driver of his power wheelchair and has a special interest in cars (and definite ideas about which brand he wants to drive when he is older!).

Anga was born a year after Ayabonga in 2005, and when he was one year old, he was diagnosed with cerebral palsy (damage to the growing brain). He has spastic diplegia, meaning his lower body is more affected than his upper body. Anga is a determined child who is independent in self-care tasks and loves running around and playing soccer – albeit with an unsteady gait.

Our rehabilitation team first met Ayabonga and Anga early in 2008, when they were four and three years old and this paper describes their journeys until they were eight and seven years old, respectively. At the time of first meeting them, they were both enrolled at an NPO-run special care centre for children with severe physical and/or intellectual disabilities in the township where they lived.

This township is a vibrant, largely Xhosa speaking community. It is a low socio-economic semi-formal settlement on the outskirts of Cape Town, with a mixture of formally built and informally constructed dwellings (or ‘shacks’), serviced mainly by communal taps and toilets. There is a mixed infrastructure of some formal roads and informal tracks, and although there is no formal rail or bus service, a regular but informal taxi service (using 12-, 16- or 21-seater minibuses) transports residents to the surrounding areas. There is one primary school, several formal and informal crèches or preschools, a community hall, two play parks, several churches, a primary health care clinic and several small ‘spaza’ shops selling basic food items and ‘shebeens’ that sell liquor. Many of the crèches, churches, spaza shops and shebeens are not registered – and even resident numbers and demographics are not formally known.

Early assessments and team reflections indicated that both children had the intellectual capacity for more formal education. However, given their under-resourced circumstances, together with their physical and to a much lesser degree learning challenges, it was felt that they would benefit most from first obtaining a solid foundation in the supportive and specialised environment of the special care centre until they reached the age of six and five years respectively (Grade R age^3^). During the subsequent two years, both children became more independent in feeding, toileting, communicating effectively and interacting confidently with their peers and carers. The therapists also provided guidance in terms of appropriate therapeutic physical activities and a graded school-readiness programme implemented by the centre’s staff and volunteers. This included activities such as cutting, drawing and perceptual activities. Ayabonga also needed a spinal brace and a power wheelchair while Anga needed ankle foot orthoses (AFOs). The therapists facilitated the provision of these assistive devices for independent mobility and correct positioning through State and other resources. Throughout this process the parents were involved and kept up to date about the children’s progress, through meetings and written reports. They were also encouraged to continue any work done at the centre during holiday times to maintain the continuity of progress made.

At the same time, the therapists continued to build relationships with the local mainstream school and local schools for Learners with Special Educational Needs (LSEN), by their broader programme of presenting therapeutic groups and treating learners identified as needing therapy. The teacher on our team also presented a programme she had developed to the teachers at the local mainstream primary school, aiming to increase understanding and acceptance of learners with special needs. The programme comprises lesson plans focussed on a range of special needs, graded from Grade 1 to Grade 7, and can be used as part of the Life Orientation learning area for each grade.

A year later, multidisciplinary evaluations by various parties once more confirmed that the children should attend a mainstream Grade R. The children and their families visited several recommended centres to look at the available mainstream pre-school options. The parents were initially hesitant about moving their children away from the security of the special care centre as they were worried about discrimination in the new placement and the possibility of decreased therapy and care. These issues were addressed in discussions and through the commitment to an on-going support system for the children, and so the parents agreed to the move, and both children were enrolled in a Grade R class at a local mainstream preschool in January 2010.

The transition to this particular pre-school was eased by firstly, the school’s commitment to include children with special needs and secondly, their previous experience of doing this successfully with two girls with special needs supported by the therapists of The Chaeli Campaign. In order to prepare and support teachers for the arrival of the two boys, discussion meetings were held in which detailed information was shared about the children’s respective conditions, about their strengths and difficulties and in which areas they could expect the children to cope with at the level of their peers, and where they needed to be sensitive to special needs and extra help needed. To enable Ayabonga to be independently mobile at school, the school had to be made physically accessible for his power wheelchair. For example, the therapists made recommendations and the school sourced funding through the local branch of Rotary International. A builder was contracted to build a ramp for access to the main entrance and additional pathways were made for wheelchair access to the playground. In addition to these environmental adaptations, which will have long-term implications for including learners far beyond Ayabonga’s current needs, the teachers were assisted with practical issues relating to the correct use and care of the two children’s specific assistive devices.

The therapists initially visited the children at the preschool weekly for therapy and once it was ascertained that the teachers and peers were managing to include the children in all activities, therapy visits were reduced to monthly. The teachers and peers came up with some workable solutions as challenges arose; for example, they moved Ayabonga’s class to the biggest available area (previously the dining room) where he had more space to manoeuvre his wheelchair; made use of a community volunteer to assist Ayabonga to do toilet transfers and moved Anga to Ayabonga’s class when he struggled being away from his friend. The school had the reassurance that the therapists were available for support between visits and to assist on an on-going basis. Such collaborative problem-solving included suggesting that Anga sit near the wall during ring time to help him get up independently off the floor afterwards and to make use of a peer to assist Ayabonga to finish cutting tasks rather than the teacher having to do this. When the staff expressed concern about the two children being teased about their disabilities, The Chaeli Campaign teacher was able to assist the class teacher’s efforts by facilitating an experiential session with the children, specifically addressing the diversity of the children in the class and encouraging acceptance of differences. Both boys managed well in Grade R and during the year they and their parents were again supported to investigate their options for Grade 1 the following year, and to find the school that would best meet the needs of each child as well as those of their families.

The collaboration with resource persons, such as teachers and other parents of children with disabilities from within the community was on-going (as also described by Karangwa, Miles and Lewis 2010). An additional milestone event at this time was The Chaeli Campaign’s employment of a community worker from the area. Living in the same community, and being the mother of a little girl with cerebral palsy herself, she was able to give crucial ‘insider’ support to the children, their parents and the schools – especially in answering questions, language translation (between Xhosa and English), providing explanations in this cross-cultural situation, and addressing issues as they arose in the times between therapists’ visits.

### Outcomes

It was initially envisioned that Ayabonga would go to his local mainstream primary school, possibly with a facilitator, but his mother chose to send him to the nearest LSEN school with boarding facilities catering specifically for children with physical disabilities. This was due to a number of factors relating to his particular circumstances, including his high physical care needs and fears of him not being accepted and included in his local mainstream primary school due to community beliefs and attitudes, his mother’s long working hours and limited support in the afternoons. The Chaeli Campaign therapist assisted with making the application to the LSEN school, accompanying them to the interview assessment, getting Ayabonga and his power wheelchair to school on his first day and taking his ‘first day at school’ photograph. She and the community worker continued to support Ayabonga and his mom through a monthly parent support group held by the community worker, being a link between the school and home when necessary and fetching him from school twice a year, which gives his family occasional breaks from the costly and lengthy trip to fetch him at weekends and provides an opportunity for the therapist to follow up with the multidisciplinary team caring for him at school. Although Ayabonga and his mom have adjusted to him being a boarder and he is receiving specialised services, he has struggled being away from home and being taught in a second language, the combination of which has led to his teacher recommending that he repeat Grade 1, to which his mother agreed. He has subsequently had a much easier start to his second year at the school and proudly talks about everything he is learning.

Anga’s family chose to send him to the local primary school, where he was enrolled into a Grade 1 class despite being on the young side. A challenging, culturally determined event occurred at the beginning of his school year due to a combination of cultural beliefs and a recent popular movie. A rumour started that he could turn himself into a snake because of the way he walks, which led to some parents requesting that the principal remove him. Such beliefs and attitudes are not uncommon in the local Xhosa culture and may be related to the more widespread cultural metaphor among other indigenous cultures in southern Africa, pertaining to an ‘internal or invisible snake’ which in turn is central in the ‘pollution beliefs’, that is, the causes of disease and modes of transmission or contagion (Green 2004). It is beyond the scope of this paper to delve deeper into this as yet little documented field, but Green’s hypothesis (2004) about the relationship between the snake beliefs and the beliefs of inner pollution and possible contagiousness, highlights the urgency of the need to understand and moreover meet the fears of these parents, that Anga’s disability could be contagious.

The Chaeli Campaign community worker, herself a member of the community and familiar with some of these beliefs, was able to attend the emergency meeting at the school to support his mother and to advocate for Anga. The therapist subsequently met with the principal and teacher to again discuss his disability and arranged to spend time with his whole class to talk about being accepting of each other’s differences and to assist with ways to help Anga and others with different challenges to be included at school through fun activities and peer-to-peer learning (e.g. experience being blindfolded, trying to walk and get up from the floor with one’s legs tied together). Allowing Anga to choose who would attend his ‘exercise’ sessions with him during the first few months of the year initially served as an incentive for peers to want to belong to his circle of friends. Anga’s mother was supported through individual visits and the monthly parent support groups run by the community worker. She has become much more assertive about the rights of those with disabilities and has told her story in various forums, including offering to do it at the school. We have also requested that the school allow The Chaeli Campaign teacher to re-launch the programme she had introduced to them a few years earlier in an attempt to avoid such discrimination occurring in the future for Anga or any other child. As he became accepted at school, we were able to decrease the frequency of our visits, and due to his resilience and love of learning, Anga successfully completed Grade 1. He then had a good start in Grade 2 with only an initial introductory visit by the therapist, the community worker and his mother needed. This was to brief his teacher about his condition and make negotiations as to a suitable time for him to be seen for bimonthly therapy.

#### Reflections

In South Africa, we have made some progress towards achieving the objectives of inclusive education as set out by White Paper 6 (Department of Education 2001). Attitudes, behaviours, teaching methods and curricula needed to be addressed, focussing on Ayabonga and Anga’s individual strengths and needs in order to maximise participation and to minimise barriers to learning. This is in line with the inclusive education principles of meeting every child’s need within the education system and acknowledging the differences in the ways in which children learn.

It has been wonderful to see these two young children settle into an environment where they are confidently learning with their peers. The process of getting these children to where they are today has been a dynamic one with many detours, and it has been vital for all parties to remain flexible and open while working towards the common goal of doing what is best for the individual child in the context of their family. It was essential to provide support for the children, their families, their current centre/school and the schools they would most likely attend in the future. The hope was to reinforce the educational process by determining, supporting and sustainably meeting each of the two children’s special needs. This included the supportive monitoring of group dynamics and social interaction throughout the process.

This process highlighted the importance of on-going parental involvement; it was important to recognise the diverse ideas on what is best for the child, to respect each person’s part in the decision-making process and to arrive at a consensus. Therapists became consultants in a more trans-disciplinary manner and the community worker was pivotal in facilitating communication and decision making, understanding the complexities faced by the families on an experiential level. The awareness of the child being first and foremost part of the family and then part of the community and other social networks, including peers and the families of peers (Garbarino & Ganzel 2000; Pillay & Di Terlizzi 2009), facilitated a more permanent change for Ayabonga’s family. So, for example, whilst we would not have chosen an English medium school for a Xhosa speaking child, Ayabonga’s family felt they needed the support of a boarding facility.

Furthermore, teachers received support and felt that they were not alone in the task of including the children. When problems arose that were new to them, they could call on the skills of the wider team (community worker, family and professionals) and this supported the sustainability of including these children.

## Recommendations

In terms of clinical practice, recommendations for professionals in the field include the prioritisation of individualised approaches and parent involvement and parent choices by facilitating collaborations. These collaborations need to be on-going and supportive and need to include the family, the child, the community, the schools (both present and future) – and to strive towards trans- or interdisciplinary approaches by professionals. The identification and support of locally-based community workers by clinicians is a key recommendation to meet the locally specific and diverse needs and to facilitate community-based approaches.

In terms of policy and curricula, it is recommended that information, skills training and advocacy issues about disabilities and their implications need to be included more formally in the curriculum for teacher training and in schools. This should aim to assist teachers and learners to include learners with disabilities in the academic as well as the social aspects of school life.

In terms of research, there is a need for the development of a sound evidence base of best practice regarding the nature and format of disability-related teaching materials for teacher and peer training, and doable ways of including these into the present school curriculum. There is also a need for more participatory methodologies where parents and teachers drive research questions in line with their needs.

Finally, in addition to the grassroots identification and support of locally based community workers described above, the formal establishment of more positions, appropriate training and support for community workers is a cross-cutting recommendation and is a step towards scaling up such an intensive programme. It requires (a) research to quantify and further substantiate the need for community workers, (b) policies to validate and reinforce this need and (c) more formalised yet practical training of community workers, including support from rehabilitation professionals, to realise this strategic aspect of developing such an intensive programme to provincial or national scale and beyond.

## Footnotes

1. In South Africa, schools for learners with special educational needs are increasingly referred to as LSEN schools. This new terminology encompasses respectful attempts to get away from the previously used, politically loaded term, ‘Special Schools’. While it may appear as a mere replacement of terminology, the new term does not carry as much stigma in the local context.

2. For more information please visit: www.chaelicampaign.co.za

3. In South Africa, Grade R (or ‘Reception’) is a year of preparation for Grade 1. While not compulsory by law yet, Grade R classes are sometimes attached to formal nursery or pre-schools, and sometimes to primary schools.
